# Perceived stress, coping behaviors, and environmental–relational stressors during first clinical placement among nursing students

**DOI:** 10.3389/fpsyg.2026.1870065

**Published:** 2026-07-15

**Authors:** Abdelaziz Hendy, Rasha Kadri Ibrahim, Amani Darwish, Nada Alqarawi, Bander Saad Albagawi, Yasir S. Alsalamah, Lisa Babkair, Nasiru Mohammed Abdullahi, Ahlam B. Alenzi, Monerh Abdullah Alfalaij

**Affiliations:** 1 Department of Maternal and Child Health Nursing, College of Nursing, Qassim University, Buraydah, Saudi Arabia; 2 Pediatric Nursing, Faculty of Nursing, Ain Shams University, Cairo, Egypt; 3 Nursing Services Department, Qassim University Medical City, Buraydah, Saudi Arabia; 4 Nursing Department, Fatima College of Health Sciences, Al Dhafra, Madinat Zayed, United Arab Emirates; 5 Pediatric Nursing, Faculty of Nursing, Damnhour University, Cairo, Egypt; 6 Psychology Department, Fatima College of Health Sciences, Abu Dhabi, United Arab Emirates; 7 Department of Community, Psychiatric, and Mental Health Nursing, College of Nursing, Qassim University, Buraydah, Saudi Arabia; 8 Medical Surgical Department, College of Nursing, Imam Mohammed Ibn Saud Islamic University, Riyadh, Saudi Arabia; 9 Department of Nursing, Mental Health Hospital, Qassim Health Cluster, Buraydah, Qassim, Saudi Arabia; 10 Faculty of Health Science, Department of Nursing, University of Pretoria, Pretoria, South Africa; 11 Faculty of Nursing, King Abdulaziz University, Jeddah, Saudi Arabia; 12 Advance Nurse Practitioner, Prince Abdullah bin Musaed Cardiac Centre (PAMCC), Arar, Northern Borders Region, Saudi Arabia; 13 Department of Emergency, Nursing Affairs, King Saud University Medical City, Riyadh, Saudi Arabia

**Keywords:** adaptation, psychological, clinical clerkship, education, nursing, clinical training environment stressors, stress, students

## Abstract

**Background:**

The transition to the first clinical placement is a psychologically demanding phase for nursing students, requiring adaptation to patient care responsibilities, academic expectations, and unfamiliar clinical settings. Stress during this period may be influenced by academic, clinical, environmental, and relational stressors, which can affect students’ psychological well-being and coping behaviors.

**Objective:**

This study aimed to assess the levels and sources of perceived stress and examine the relationship between coping behaviors and environmental–relational stressors among first-year nursing students during their initial clinical placement.

**Methods:**

Design: Cross-sectional descriptive correlational study. A total of 467 first-year undergraduate nursing students undergoing initial clinical training in seven governmental hospitals participated. Data were collected using the Perceived Stress Scale for Nursing Students and the Coping Behavioral Inventory. The Perceived Stress Scale included domains related to the clinical environment and interactions with teaching and nursing staff, which were used to explore perceived environmental and relational stressors. Descriptive statistics, Pearson correlations, and multiple linear regression were used to examine associations between stress domains and coping behaviors.

**Results:**

Students reported high overall perceived stress (*M* = 62.99, SD = 9.76). The most prominent stressors were practical assignments and workload, patient care, examinations of personal competence, and environmental and staff-related factors, while stress related to lack of professional knowledge/skills and daily life was comparatively lower. Regarding coping behaviors, problem-solving and optimistic coping showed significant negative associations with total perceived stress (*r* = −0.75 and *r* = −0.36, respectively; *p* < 0.001) and were significant negative predictors in regression analysis (*β* = −0.76 and *β* = −0.32; *p* < 0.001).

**Conclusion:**

First-year nursing students experienced high stress during initial clinical placement, mainly related to workload, patient care, evaluation, and environmental factors. Coping strategies showed associations with stress levels, where problem-solving and optimism were linked to lower perceived stress, while avoidance and transference showed weaker associations. These findings highlight the potential importance of structured orientation, mentorship, stress-management support, and supportive clinical training conditions during early clinical exposure.

## Introduction

Clinical practice is a central component of nursing education, enabling students to apply classroom knowledge, develop technical and professional competencies, and build professional relationships in real patient care settings (Mohamed et al., 2024). However, the transition to clinical training can be stressful for nursing students because it requires adaptation to academic expectations, practical responsibilities, and direct patient care (Ibrahim et al., 2024). Stress may increase as students encounter more complex clinical tasks and are expected to integrate theoretical knowledge with professional performance under supervision (Suheir and Sayed, 2022; Dunn and Burnett, 1995).

For first-year nursing students, the initial clinical placement represents a particularly demanding transition because students are exposed to unfamiliar clinical routines, direct patient-care responsibilities, workload demands, and evaluation by instructors and clinical staff. These experiences may generate stress related not only to clinical tasks but also to environmental and relational aspects of the placement setting (Chan, 2002; Marques et al., 2021; Masso et al., 2022).

Early clinical experiences are important to examine because they can influence students’ learning, professional development, confidence, and retention in nursing education (Dias et al., 2024; Byron, 2018). During clinical training, students are expected to apply theoretical knowledge, make decisions, communicate with patients and staff, and manage patient-care responsibilities under supervision. Evidence indicates that nursing students commonly experience moderate-to-high levels of stress during clinical rotations, which may affect psychological well-being, learning engagement, and skill development (Labrague, 2024; Welch, 2023).

Clinical stress refers to the perceived imbalance between clinical demands and students’ available coping resources. Among nursing students, stress may arise from patient-care responsibilities, clinical examinations, workload, academic assignments, limited preparedness, and interactions with clinical personnel. These stressors can affect students’ emotional well-being, academic engagement, and ability to adapt to clinical training. Therefore, understanding both the sources of stress and students’ coping responses is essential for developing supportive educational interventions (Alshowkan, 2022; Visier-Alfonso et al., 2024; Reverté-Villarroya et al., 2021).

Clinical stress among nursing students should be understood not only as an individual psychological response but also as clinical training environment stress shaped by environmental and relational stressors within the clinical placement setting (Dias et al., 2024; Labrague, 2024). During initial clinical placement, students encounter unfamiliar patient-care responsibilities, workload demands, evaluation pressure, and interactions with teaching and nursing staff, all of which can intensify perceived stress and shape coping behaviors. In this study, these environmental–relational stressors were examined through students’ perceived stress responses, particularly the Perceived Stress Scale domain related to the clinical environment and interactions with teaching and nursing staff, rather than as a separate multidimensional clinical learning environment construct (Aryuwat et al., 2024; Jamshidi et al., 2016).

Coping strategies refer to the cognitive and behavioral efforts individuals use to manage stressful situations (Kaur et al., 2020; Jawabreh, 2024). In nursing education, coping behaviors are especially important because they shape how students respond to academic demands, clinical responsibilities, and stressful interactions in practice settings (Visier-Alfonso et al., 2024; Efstathiou et al., 2025). Problem-solving is an active coping strategy that involves identifying solutions, planning responses, and taking direct action to manage stressors. Optimism reflects a positive orientation toward stressful situations and supports more adaptive adjustment during demanding clinical experiences (Jadoon et al., 2023).

In contrast, avoidance coping involves withdrawing from, delaying, or avoiding direct engagement with stressors and is often associated with less adaptive psychological outcomes (Goh et al., 2010; Biggs et al., 2017). Transference coping refers to redirecting attention or emotional responses away from the stressor through activities such as rest, entertainment, or other forms of emotional release (Kpeno et al., 2024). Although these coping strategies may provide short-term relief, they are likely to reflect different ways of responding to ongoing clinical demands. Examining both active and passive coping behaviors therefore offers a more comprehensive understanding of how nursing students manage and respond to stress during their initial clinical placement (Stanisławski, 2019; Palacios-Delgado et al., 2024; Theodoratou and Argyrides, 2024; Ibrahim et al., 2025).

Despite growing evidence on stress among nursing students during clinical practice, limited attention has been given to how different stressor domains relate to coping behaviors during the first clinical placement (Masso et al., 2022; Wan Mamat et al., 2023; Aryuwat et al., 2024; AlAzri et al., 2023; Jamshidi et al., 2016; Bhurtun et al., 2019; Bhandari and Gurung, 2023; Kahriman et al., 2024; Rafati et al., 2021). In particular, less is known about the relationship between environmental and relational stressors within the clinical training environment and students’ use of active or passive coping strategies across different educational contexts.

The Transactional Model of Stress and Coping was used as the theoretical framework for this study. The model conceptualizes stress as a dynamic process arising from the interaction between individuals and their environment, in which perceived demands are appraised in relation to available coping resources (Lazarus and Folkman, 1984). This framework is appropriate for examining first-year nursing students’ initial clinical placement because it explains how students appraise clinical demands, environmental–relational stressors, and evaluation pressures in relation to their available coping responses. Applying this model facilitates the examination of perceived stress domains and coping behaviors and provides a basis for identifying educational strategies to support students during early clinical exposure.

### Aim

This study aimed to assess the levels and sources of perceived stress and examine the relationship between coping behaviors and environmental–relational stressors among first-year nursing students during their initial clinical placement.

## Methods

### Study design

This study employed a cross-sectional, descriptive, correlational design using a quantitative approach to examine the relationships between Stress and Coping Behaviors in Nursing Students During Initial Clinical Practice. This study was reported in accordance with the STROBE (Strengthening the Reporting of Observational Studies in Epidemiology) guidelines for cross-sectional studies

### Setting

The research was conducted in seven hospitals in Cairo, Egypt, comprising a mix of governmental and university-affiliated healthcare institutions. These hospitals are officially affiliated with the nursing faculty and are the main clinical teaching hubs for undergraduate nursing students. In these hospitals, students are assigned to various wards and learn to provide patient care. The clinical training aligns with the nursing program’s academic schedule and is conducted by faculty members and clinical mentors who supervise students. The training is designed to provide students with the requisite clinical skills while incorporating theoretical components. Faculty members supervise students using standard evaluation instruments, including checklists, structured observations, and other methods to assess knowledge, skills, attitudes, and competencies. Furthermore, all the governmental and university hospitals that participated in the research provide students with a wide range of patients, complex cases, and varied working conditions, facilitating a rich learning experience. This diversity ensures that nursing students are exposed to a range of relevant study-related stressors and coping challenges.

### Subjects

A convenience sampling technique was used to recruit first-year undergraduate nursing students enrolled in the Faculty of Nursing. The study included 467 students from seven affiliated governmental hospitals where students received their initial clinical training during the first academic semester. This clinical placement represented the students’ first exposure to real hospital environments and direct patient care experiences. Both male and female students were included according to the actual demographic distribution of the nursing program.

Eligible participants were students officially registered in the first semester who attended the scheduled initial clinical training sessions during the data collection period. Students were approached directly by the researchers during clinical training sessions, informed about the study’s purpose and procedures, and invited to participate voluntarily. Those who agreed completed the self-administered questionnaires anonymously. Students who were absent during data collection or declined participation were excluded from the study.

All participating hospitals were affiliated with the same Faculty of Nursing and followed a standardized curriculum for first-year clinical training. Students were exposed to comparable learning objectives and supervised clinical practice; however, minor variations in clinical workload, patient case mix, and ward environment may have existed between sites. The inclusion of students from multiple clinical training hospitals enhanced the diversity and representativeness of the sample among first-year nursing students undergoing initial clinical exposure at the participating institutions. However, because convenience sampling was used, the generalizability of the findings to similar educational and clinical contexts should be interpreted with caution.

Of 539 eligible students, 467 completed the questionnaire (response rate 86.6%). Due to the anonymous nature of data collection, non-responders could not be formally compared with responders; however, the high response rate suggests a low risk of substantial non-response bias.

### Sample size

As reported by Mohamed et al. in 2024, 52.5% of first-year nursing students experienced moderate-to-severe stress (Mohamed et al., 2024). This figure was used to compute the sample size for the study utilizing Cochran’s formula for a single proportion (Lwanga et al., 1991). A 95% confidence level (*Z* = 1.96), a population proportion (p) of 0.525, and a 5% margin of error (*d* = 0.05) were used, yielding a minimum required sample size of 384 students. To account for no responders, a 10% increase was added, raising the sample requirement to 427 participants. A total of 467 students participated in the current study, exceeding the calculated minimum sample size and thereby improving the statistical precision and reliability of the findings.

### Tools

#### Characteristics of students

Age, gender, previous participation in caring for a sick family member, Reason for enrollment in the Faculty of Nursing, having suffered from a chronic disease, and any family member who has previously studied nursing.

#### Coping behavioral inventory scale (CBI)

Coping behavior refers to the strategies people use to handle stressful situations. In this study, students’ coping behaviors were assessed using the Coping Behavioral Inventory Scale (CBI), originally developed by Sheu et al. (2002). The tool includes 19 items, each rated on a 5-point Likert scale ranging from 1 (never) to 5 (always). The items are grouped into four categories: problem-solving actions (6 items), such as making plans and setting goals to solve problems; optimistic coping actions (4 items), such as keeping a positive outlook and believing in one’s ability to overcome challenges; avoidance actions (6 items), such as avoiding teachers or attributing problems to fate; and transference actions (3 items), such as relaxing through sleep, entertainment, or exercise. Higher scores in each category indicate more frequent use of that coping strategy. The original validation of the CBI showed that the four factors explained 38.2% of the total variance, with a Cronbach’s alpha of 0.76, suggesting moderate reliability (Sheu et al., 2002). Also, in a recent study, the Cronbach’s alpha for CBI was 0.88 (Toqan et al., 2023). The Cronbach’s alpha test for the current study was 0.87, confirming that the scale was reliable for use with our sample.

#### Perceived stress scale (PSS)

To measure students’ perceived stress during clinical practice, the Perceived Stress Scale (PSS) developed by Sheu et al. (2002) was used. This version, adapted specifically for nursing students, consists of 30 items rated on a 5-point Likert scale, from 0 (not stressful at all) to 4 (very stressful). The items cover six areas of stress: lack of professional knowledge and skills (9 items), practical assignments and workload (8 items), taking care of patients (4 items), examinations of personal competence (3 items), environment and teaching staff (4 items), and daily life stressors (2 items). The total score ranges from 0 to 120, with higher scores indicating greater stress. The PSS has well-established psychometric properties. In Sheu et al.’s original study, Cronbach’s alpha was 0.89, and the content validity index was 0.94 (Sheu et al., 2002). Also, Jimenez et al. (2010) reported that the internal consistency of the scale was excellent (Cronbach’s alpha = 0.92), and the Spearman–Brown split-half reliability was 0.80 (*p* < 0.001). The Cronbach’s alpha test for the current study was 0.91, confirming that the scale was reliable for use with our sample.

### Fieldwork

Recruitment was conducted uniformly across the seven participating hospitals. First-year nursing students were approached during their scheduled clinical training sessions and invited to participate. A standardized explanation of the study purpose, eligibility criteria, and ethical considerations was provided to all participants. Data collection followed a consistent protocol across all sites using the same online questionnaire.

In our study, data were collected via an online survey administered to students at various healthcare facilities. The online approach was adopted to maximize access while safeguarding participant identities and facilitating rapid data collection across different hospital wards and regions. A short message inviting participants to take part in the study, containing its objectives, inclusion criteria, confidentiality guarantees, and ethical considerations, was placed at the start of the survey.

The study instruments were administered in English, as English is the official language of nursing education and clinical training for the participating students. All students were able to read and understand English adequately. Prior to data collection, the questionnaires were pilot-tested on a small group of nursing students (35 students) to ensure clarity and comprehensibility of the items. These students were excluded from the final study sample and data analysis.

The online survey contained custom-made, tested tools to measure the Coping Behavioral Inventory Scale and the Perceived Stress Scale, as well as capturing demographic information relevant to the participants. The survey was open for responses for [6 weeks], and reminders were sent periodically to encourage participation. All responses were automatically recorded and securely stored in a database protected by a password known only to the research team. This reduces the need for manual capture operations, which are prone to mistakes, while enabling timely statistical analysis.

#### Ethical consideration

Ethical approval was granted by the IRB of MTI University, located in Cairo, Egypt. The IRB approval number is 143/2025. This study adhered to the ethical principles of the Declaration of Helsinki, and all students received appropriate information about the study’s purpose and scope, as well as its potential benefits. The researchers underscored the voluntary nature of the investigation and made it clear that students could withdraw from the study without any consequences.

Informed consent was obtained electronically, and participation was voluntary. The survey was conducted online using Google Forms. Participants received the survey only after checking the consent box. Students could withdraw from the study at any point without facing consequences. The data encoded to maintain confidentiality ensured that no identifiable information was leaked.

#### Statistical analysis

The data were examined using the Statistical Package for the Social Sciences (SPSS, Version 26). For all study variables, basic descriptive statistics, including means and standard deviations, were computed. The Kolmogorov–Smirnov test and the Shapiro–Wilk test was used to evaluate the normality of continuous variables. Prior to conducting the regression analysis, the assumptions of multiple linear regression were evaluated. Multicollinearity among independent variables was assessed using tolerance and variance inflation factor (VIF) values, which were within acceptable ranges. Residuals were also examined for normality, linearity, homoscedasticity, and independence to ensure the appropriateness of the regression model. Because the study relied on self-reported questionnaires collected at a single time point, the findings were interpreted as associative rather than causal relationships. Results indicated that the distributions did not significantly deviate from normality, with Kolmogorov–Smirnov values ranging from *D* = 0.041 to 0.067 (*p* = 0.20–0.28) and Shapiro–Wilk values ranging from *W* = 0.978 to 0.991 (*p* = 0.12–0.25). As all *p*-values were greater than 0.05, parametric tests were appropriate.

To study the relationships between the coping strategies (avoidance, problem-solving, optimism, and transference) and the domains of stress (knowledge and skills, workload, patient care, examinations, environment and staff, and daily life), as well as with total stress, Pearson’s correlation coefficients (r) were calculated. Separate multiple linear regression analyses for each outcome variable were performed with stress levels as the dependent variable. The coping strategies and demographic variables (age, gender, being a former medical student, personal interest in nursing, history of caring for a sick family member, and chronic illness) were entered as predictor variables. Multicollinearity was assessed using variance inflation factor (VIF) values; all were under 2, indicating no serious multicollinearity. The threshold of statistical significance was established at *p* < 0.05, measured bidirectionally.

## Results

Table 1 presents the demographic and background characteristics of the studied nursing students (*n* = 467). The mean age was 19.76 years (SD = 0.98). The majority of participants were female (65.7%), while 34.3% were male. Only a small proportion of students (5.8%) had previously participated in caring for a sick family member. Regarding the reasons for enrollment in the Faculty of Nursing, nearly three-quarters (72.6%) reported that admission was determined by coordination grades, whereas 27.4% indicated personal desire. A small minority of students (4.1%) reported suffering from a chronic disease. In addition, 15.2% of students reported having a family member who had previously studied nursing, while 84.8% did not.

**Table 1:** Characteristics of studied nursing students (n = 467).

Characteristics	*n*	%
Age		
Mean (SD) 19.76 (0.98)		
Gender		
Male	160	34.3
Female	307	65.7
Previously participated in the caring of a sick family person		
Yes	27	5.8
No	440	94.2
Reason for your enrollment in the Faculty of Nursing		
Personal desire	128	27.4
Coordination grades	339	72.6
Suffered from chronic disease		
Yes	19	4.1
No	448	95.9
Family member with nursing background		
Yes	71	15.2
No	396	84.8

Table 2 shows the mean scores, standard deviations, and interpretations of coping strategies and stress among nursing students (*N* = 467). Among the coping strategies, avoidance (*M* = 17.37, SD = 4.64; 57.9%) and transference (*M* = 7.64, SD = 1.49; 50.9%), whereas problem-solving (*M* = 8.9, SD = 2.75; 29.7%) and optimistic coping (*M* = 6.65, SD = 1.42; 33.2%). Regarding perceived stress, clinical stressors such as practical assignments and workload (*M* = 18.14, SD = 4.49; 56.7%) and taking care of patients (*M* = 10.24, SD = 2.51; 64%), while lack of professional knowledge and skills (*M* = 14.05, SD = 2.27; 39.1%). Academic stressors, including examinations of personal competence (*M* = 6.82, SD = 1.56; 56.8%) and environment/teaching and nursing staff (*M* = 10.36, SD = 2.07; 64.8%). In contrast, external stressors such as daily life stress (*M* = 3.38, SD = 0.49; 42.3%). Overall, the total stress score was *M* = 62.99 (SD = 9.76; 52.5%), indicating that nursing students experienced considerable stress during their academic and clinical training.

**Table 2:** Mean and SD of coping strategy and stress among nursing students (*n* = 467).

Domains	Mean	SD	Mean percent
Avoidance	17.37	4.64	57.9%
Problem-solving	8.9	2.75	29.7%
Optimistic	6.65	1.42	33.2%
Transference	7.64	1.49	50.9%
Total perceived stress scale	62.99	9.76	52.5%
Clinical stressors			
Lack of professional knowledge and skills	14.05	2.27	39.1%
Practical assignments and workload	18.14	4.49	56.7%
Taking care of patients	10.24	2.51	64%
Academic stressors			
Examinations of personal competence	6.82	1.56	56.8%
Environment and teaching and nursing staff	10.36	2.07	64.8%
External stressors			
Stress from daily life	3.38	0.49	42.3%

According to the correlation analysis, coping strategies were significantly associated with various stress domains among nursing students. Specifically, problem-solving coping demonstrated the strongest negative correlations with stress across domains, including lack of professional knowledge and skills (*r* = −0.66, *p* < 0.001), practical assignments and workload (*r* = −0.31, *p* < 0.001), taking care of patients (*r* = −0.38, *p* < 0.001), examinations of personal competence (*r* = −0.51, *p* < 0.001), environment and teaching and nursing staff (*r* = −0.62, *p* < 0.001), daily life (*r* = −0.12, *p* = 0.01), and total stress (*r* = −0.75, *p* < 0.001). Staying optimistic also showed significant negative correlations with examinations of personal competence (*r* = −0.27, *p* < 0.001), environment and staff (*r* = −0.42, *p* < 0.001), daily life (*r* = −0.34, *p* < 0.001), and total stress (*r* = −0.36, *p* < 0.001). In contrast, avoidance coping exhibited weak positive correlations with workload (*r* = 0.16, *p* = 0.032) and taking care of patients (*r* = 0.13, *p* = 0.038), while not significantly related to total stress (*r* = 0.10, *p* = 0.062). Transference coping was weakly and inconsistently associated, showing a small positive correlation with examinations (*r* = 0.12, *p* = 0.01), a negative correlation with daily life (*r* = −0.15, *p* = 0.02), and a non-significant negative relationship with total stress (*r* = −0.11, *p* = 0.051) (Table 3).

**Table 3:** Correlations between stress domains and coping behaviors (*n* = 467).

Domains	Lack of professional knowledge and skills		Practical assignments and workload		Taking care of patients		Examinations of personal competence		Environment and teaching and nursing staff		Daily life		Total	
	*r*	*p*	*r*	*p*	*r*	*p*	*r*	*p*	*r*	*p*	*r*	*p*	*r*	*p*
Avoidance	0.01	0.92	0.16	0.032	0.13	0.03	0.08	0.12	0.04	0.28	−0.05	0.31	0.10	0.06
Problem-solving	−0.66	0.0	−0.31	0.0	−0.38	0.00	−0.51	0.00	−0.62	0.00	−0.12	0.01	−0.75	0.00
Stay optimistic	−0.11	0.01	−0.15	0.03	−0.02	0.73	−0.27	0.00	−0.42	0.00	−0.34	0.0	−0.36	0.00
Transference	0.09	0.10	0.04	0.45	0.03	0.52	0.12	0.01	0.05	0.31	−0.15	0.02	−0.11	0.05

Table 4 presents the Pearson correlation coefficients among the four coping styles: avoidance, problem-solving, optimism, and transference. Avoidance was significantly and negatively correlated with problem-solving (*r* = −0.17, *p* = 0.01) and optimism (*r* = −0.12, *p* = 0.02). A moderate positive correlation was observed between avoidance and transference (*r* = 0.41, *p* < 0.001). A weak positive correlation was found between problem-solving and optimism (*r* = 0.10, *p* = 0.03). In contrast, correlations between problem-solving and transference (*r* = −0.04, *p* = 0.32) and between optimism and transference (*r* = −0.05, *p* = 0.36) were not statistically significant.

**Table 4:** Correlations between coping strategies (*n* = 467).

Domains	Avoidance		Problem-solving		Stay optimistic		Transference	
	*r.*	*p.*	*r.*	*p.*	*r.*	*p.*	*r.*	*p.*
Avoidance								
Problem-solving	−0.17	0.01						
Stay optimistic	−0.12	0.02	0.10	0.03				
Transference	0.41	0.00	−0.04	0.32	−0.05	0.36		

The multiple linear regression model was statistically significant, *F* (9,457) = 123.24, *p* < 0.001, indicating that coping strategies and demographic characteristics were jointly associated with perceived stress levels among nursing students. The model explained 70.8% of the variance in stress levels (*R*^2^ = 0.708, Adjusted *R*^2^ = 0.702), corresponding to a large effect size (Cohen’s *f*^2^ = 2.42).

Among the predictors, problem-solving coping (*β* = −0.76, *p* < 0.001) and optimistic coping (*β* = −0.32, *p* < 0.001) showed significant negative associations with perceived stress. Age (*β* = −0.14, *p* < 0.001) and gender (male; *β* = −0.06, *p* = 0.035) were also significantly associated with lower stress levels. In contrast, no experience of caring for a sick family member was significantly associated with higher stress (*β* = 0.08, *p* = 0.011). Other variables, including avoidance coping, transference coping, previous medical student status, and chronic disease, were not statistically significant predictors (Table 5).

**Table 5:** Multiple linear regression predicting perceived stress among first-year nursing students (*N* = 467).

Predictors	*B*	SE	*t*	*p*	*β*	VIF	CI_lower	CI_upper
Const	91.29	3.38	27.00	0.00	0.0		84.65	97.94
Avoidance	−0.02	0.06	−0.31	0.75	−0.011	1.90	−0.14	0.10
Problem-solving	−1.32	0.04	−29.02	0.00	−0.76	1.07	−1.41	−1.23
Stay optimistic	−1.06	0.08	−11.98	0.00	−0.32	1.08	−1.24	−0.89
Transference	−0.14	0.10	−1.43	0.15	−0.04	1.50	−0.34	0.05
Age	−0.85	0.16	−5.34	0.00	−0.14	1.13	−1.16	−0.54
Gender: “Male”	−0.58	0.27	−2.11	0.03	−0.06	1.05	−1.12	−0.04
Family member with nursing background: “yes”	−0.60	0.36	−1.66	0.09	−0.04	1.08	−1.32	0.11
Care sick family “No”	1.51	0.59	2.54	0.01	0.08	1.34	0.35	2.67
Chronic disease “No”	0.62	0.54	1.15	0.25	0.03	1.13	−0.44	1.69

*R*^2^ 0.708, Adjusted *R*^2^ 0.702, effect size 2.42, *F* = 123.24, *p* < 0.001.

## Discussion

This study examined the levels and sources of perceived stress among nursing students during their initial clinical practice and explored their coping behaviors, including problem-solving, optimism, avoidance, and transference. The findings indicated a high overall level of perceived stress, with stress related to the clinical training environment particularly environmental and staff-related factors associated with interactions with teaching and nursing staff being among the most prominent sources. Patient care, practical assignments, workload, and examinations of personal competence were also identified as important stressors, whereas stress related to a lack of professional knowledge and skills, as well as daily life, was comparatively lower.

The findings suggest that stress among first-year nursing students is not limited to individual academic or clinical demands but may also be associated with relational and contextual factors within the clinical placement setting. In particular, interactions with teaching staff and nursing personnel may influence students’ perceived confidence, emotional adjustment, and willingness to seek support. Students who perceive the clinical environment as highly evaluative, unsupportive, or intimidating may also report greater hesitation in asking questions, expressing uncertainty, or requesting guidance, alongside higher levels of perceived stress and reduced engagement in learning activities.

These results also point to the importance of institutional support during early clinical exposure, including adequate orientation, structured supervision, clear expectations, and supportive guidance from clinical instructors and nursing staff. The reliance on avoidance and transference coping observed in this study may reflect students’ responses to clinical environments perceived as insufficiently supportive rather than solely individual coping preferences. Furthermore, these findings may be partially explained by the influence of the hidden curriculum, where implicit norms, expectations, and behaviors within clinical settings shape students’ experiences. Negative role modeling, hierarchical communication, and fear of evaluation may be associated with increased perceived stress and reduced student engagement, even in contexts where formal curricula emphasize supportive learning environments. Overall, these findings suggest that nursing students’ stress experiences are shaped not only by workload and clinical responsibilities but also by environmental and relational factors within the clinical training context. In particular, interactions with teaching and nursing staff appear to represent important sources of stress during the first clinical placement. These findings mirror results from prior studies in different settings where a significant proportion of nursing students in their first clinical experience reported high total perceived stress (Ebrahim et al., 2024; Almutairi, 2024; Salvarani et al., 2020; Benerji et al., 2024), high stress from staff (McCarthy et al., 2018; Reddy et al., 2018), high practical assignments and workload (Ching et al., 2020), and low stress levels from personal sources (Vea et al., 2024).

This may be related to multiple factors, particularly the dual demands placed on nursing students during their academic and clinical training. The high overall perceived stress reflects the ongoing need to balance theoretical learning with practical application in real clinical settings. Environmental factors, teaching approaches, and interactions with nursing staff were also among the major reported stressors, suggesting that relational and contextual aspects of the clinical placement may be associated with students’ training experiences and perceived level of support.

Furthermore, clinical tasks such as patient care, practical assignments, and workload were among the commonly reported stressors during initial clinical placement. These stressors may be associated with the emotional and physical demands of early clinical exposure as well as evaluation processes by supervisors. In contrast, stress related to a lack of professional knowledge and skills showed comparatively lower scores. This finding may indicate that students experienced greater stress when applying knowledge in real clinical situations compared with theoretical learning. In addition, the relatively lower daily life stress scores suggest that academic and clinical demands may represent more prominent sources of perceived stress within this sample.

The findings showed that nursing students relied more frequently on avoidance and transference coping strategies, whereas problem-solving and optimism were less commonly used during initial clinical placement. This pattern may reflect the challenges associated with early clinical exposure, including high levels of perceived stress, unfamiliar patient-care responsibilities, emotional demands, and concerns related to clinical evaluation. In addition, most students reported no prior experience caring for sick family members, and most did not have family members with previous nursing education, which may have limited their familiarity with clinical care environments. Furthermore, most students reported that enrollment in the Faculty of Nursing was primarily related to coordination grades rather than personal desire.

These results are anchored with study results among 786 first-year nursing students at universities in southern Poland that reported students with higher stress levels are more likely to engage in avoidance behaviors, which can hinder their learning and professional development (Bodys-Cupak et al., n.d.). A qualitative study among first-year nursing students in Sydney noted that the lack of structured support and guidance in managing emotions during clinical placements exacerbates the reliance on these coping mechanisms (McCloughen et al., 2020). A recent literature mentioned that approximately 41.9% of students reported lacking confidence, which correlates with lower engagement in problem-solving (Abdulrahman et al., 2024).

Correlation analysis showed that greater reported use of problem-solving and optimism was associated with lower levels of perceived stress among first-year nursing students. Greater reported use of problem-solving and optimism was associated with lower perceived stress among first-year nursing students. However, because the study used a cross-sectional design, these findings should not be interpreted as evidence that these coping behaviors reduce stress or function as protective factors. It is also possible that students with lower perceived stress were more likely to report problem-solving and optimism, or that other unmeasured factors influenced both stress and coping responses. The regression analysis showed that problem-solving and optimism were significantly associated with lower perceived stress. These findings indicate an association between constructive coping patterns and perceived stress levels during initial clinical placement. However, given the cross-sectional design, the directionality of this relationship cannot be established. It therefore remains unclear whether greater use of problem-solving and optimism is associated with lower stress, whether students with lower stress are more likely to report these coping strategies, or whether other unmeasured factors may account for the observed associations. Similarly, a study conducted in Qatar among medical students investigated the correlation between coping strategies, optimism, and stress, revealing that junior medical residents had low optimism ratings and elevated scores in avoidant coping styles (Smida et al., 2021).

The relationship indicated that avoidance coping was positively associated with workload and patient care, although it was not substantially linked to overall stress. Conversely, transference coping had a marginal positive link with examinations, a negative correlation with daily living, and a non-significant negative association with overall stress. Moreover, regression analysis revealed that neither avoidance nor transference coping was a significant predictor of stress; however, the correlation results showed a moderate and significant positive association between the two. Avoidance and transference showed weaker and less consistent associations with perceived stress across domains. These coping behaviors may reflect students’ responses to stressful clinical situations; however, the present study cannot determine their effectiveness or long-term impact.

Overall, the findings indicate that some nursing students reported greater use of avoidance and transference coping strategies during early clinical placement. These coping patterns showed associations with stressors related to workload, patient care, and examinations, and may reflect students’ responses to the demands and challenges of initial clinical exposure rather than fixed coping preferences.

Regarding demographic variables, older age and male gender were associated with lower perceived stress levels. In addition, students without previous experience of caring for a sick family member reported higher stress. These associations may suggest that factors such as prior exposure to caregiving situations, age-related experience, or familiarity with illness contexts could be related to variations in perceived stress during initial clinical training. However, these interpretations remain tentative, as the study did not measure related constructs such as maturity, resilience, confidence, social support, or preparedness, which may also influence stress perceptions. These findings align with a qualitative study among nursing students, which indicated that individuals who had not assumed responsibility for a critical patient had elevated levels of worry (Yıldız et al., 2025). In a comparable vein, a meta-synthesis analysis by Liu et al. identified that the primary challenge faced by nursing students during their initial exposure to the ICU was ambiguity, which subsequently resulted in psychological issues such as fear and anxiety (Liu et al., 2022).

The findings suggest that students tended to use avoidance and transference together as passive coping strategies during stressful clinical situations. In contrast, problem-solving and optimism appeared to function as more active and constructive coping strategies. This pattern is consistent with coping theory, which distinguishes between approach-oriented and avoidance-oriented coping. However, because the study used a cross-sectional design, these results should be interpreted with caution, and no conclusions can be drawn about the long-term effectiveness of these coping strategies.

### Limitations

This study has several limitations. First, the cross-sectional design prevents conclusions about causality or directionality between perceived stress and coping behaviors. Therefore, the findings should be interpreted only as associations. Second, data were collected from first-year nursing students in selected hospitals in Cairo, which may limit the generalizability of the findings to students in other academic years, institutions, or cultural contexts. Third, all data were self-reported, which may introduce recall bias or social desirability bias.

Another important limitation is that the study did not directly measure the clinical learning environment, psychological safety, relational dynamics, or the hidden curriculum using dedicated validated instruments. Instead, environmental and relational aspects were explored indirectly through stress-related domains of the Perceived Stress Scale, particularly the “environment and teaching/nursing staff” domain. Therefore, these concepts should be understood as theoretical and contextual interpretations rather than measured variables in the current study. Future studies should incorporate validated instruments, such as CLEI, CLES+T, psychological safety scales, and measures of professional identity or the hidden curriculum, to provide a more comprehensive assessment of these constructs.

In addition, the cross-sectional design limits the ability to establish causal or directional relationships between perceived stress and coping behaviors. The study relied on self-reported measures, which may increase the possibility of response bias and common method variance. Finally, the use of convenience sampling may limit the generalizability of the findings beyond similar educational and clinical settings.

The relatively high explained variance should be interpreted cautiously, as both stress and coping behaviors were measured using self-reported instruments at a single time point. This may introduce common method variance and conceptual overlap between constructs, potentially inflating observed associations. Therefore, the findings should be interpreted as correlational rather than explanatory or causal.

## Conclusion

This study demonstrated that first-year nursing students experience substantial stress during their initial clinical placement, particularly related to environmental–staff interactions, patient care responsibilities, workload, and clinical evaluation demands. Coping strategies varied among students, with problem-solving and optimism showing significant associations with lower perceived stress, whereas avoidance and transference showed weaker and more inconsistent relationships with stress across domains.

These findings suggest that different coping patterns are associated with different levels of perceived stress during early clinical exposure. However, given the cross-sectional design, no conclusions can be drawn regarding causality, effectiveness, or directional relationships between coping strategies and stress levels.

The findings highlight the importance of supportive clinical training conditions, including effective supervision, clear communication, structured orientation, and adequate institutional support during early clinical exposure. Such supportive environments may be associated with better psychological adjustment and coping patterns among nursing students. Further longitudinal and interventional research is needed to clarify how coping behaviors and clinical stress evolve over time and whether targeted interventions can improve students’ adaptation during clinical training.

### Implication of practice

The findings of this study suggest potential areas of focus for supporting nursing students during their initial clinical placement. Given the observed associations between perceived stress, coping behaviors, and clinical training environment factors, nursing educators and clinical supervisors may consider strengthening structured orientation, supervision, communication, and student support mechanisms during early clinical exposure. These suggestions are intended to reflect observed relationships rather than tested interventions.

In addition, the associations observed between coping strategies (particularly problem-solving and optimism) and lower perceived stress may inform the development of future educational strategies aimed at enhancing coping skills and stress awareness among nursing students. However, these recommendations should be interpreted as hypothesis-generating, as the current study design does not allow evaluation of intervention effectiveness or causal effects.

Future research is needed to examine these relationships using longitudinal and experimental designs in order to determine whether targeted interventions, mentorship programs, or modifications to the clinical learning environment can effectively reduce stress and improve coping outcomes among nursing students during clinical training.

## Data Availability

The raw data supporting the conclusions of this article will be made available by the authors, without undue reservation.
